# Comparative effectiveness of resistance training modalities after anterior cruciate ligament reconstruction: a network meta-analysis of randomized controlled trials

**DOI:** 10.3389/fphys.2026.1792091

**Published:** 2026-08-03

**Authors:** Jian Chen, Yuxiang Wu, Hui Xu, Yangliang Ren, Renjun Yang, Sijia Yao, Wanpei Luo, Jianping Yi, Xu He, Lin Zhang

**Affiliations:** 1 Department of Traditional Chinese Medicine, West China Hospital Sichuan University Jintang Hospital, Jintang First People’s Hospital, Chengdu, China; 2 Department of Rehabilitation Therapy, Hainan Medical University, Haikou, China; 3 Department of Orthopedics, West China Hospital Sichuan University Jintang Hospital, Jintang First People’s Hospital, Chengdu, China; 4 Department of Rehabilitation, West China Hospital Sichuan University Jintang Hospital, Jintang First People’s Hospital, Chengdu, China; 5 HeCares Integrative Medicine Center, Sunnyvale, CA, United States

**Keywords:** anterior cruciate ligament reconstruction, blood flow restriction, eccentric training, isokinetic training, network meta-analysis, resistance training

## Abstract

**Background:**

After anterior cruciate ligament reconstruction (ACLR), resistance training is essential, yet the comparative effectiveness of specific approaches remains uncertain. The objective was to compare the effectiveness of commonly used resistance training modalities after ACLR on quadriceps strength, quadriceps mass, knee function, and health-related quality of life (QoL).

**Methods:**

PubMed, Embase, Cochrane CENTRAL, Web of Science, and Google Scholar were searched to 30 September 2025 for randomized controlled trials in adults or adolescents after primary ACLR. Eligible studies compared modality-dominant resistance-based rehabilitation with standard multimodal postoperative rehabilitation or other resistance approaches and reported quadriceps strength, quadriceps mass, knee function, or QoL. The standard multimodal rehabilitation comparator was interpreted as usual rehabilitation practice rather than absence of exercise or resistance training. A random-effects network meta-analysis estimated standardized mean differences (SMDs) with 95% confidence intervals and ranked treatments using surface under the cumulative ranking curve (SUCRA).

**Results:**

Twenty-two trials (n = 797) were included. For quadriceps strength, blood flow restriction training (BFRT) ranked first (SUCRA 81.7) and exceeded standard multimodal rehabilitation (CON) (SMD 0.40; 95% CI 0.07 to 0.73). For quadriceps mass, BFRT ranked first (SUCRA 79.1) and exceeded CON (SMD 0.82; 95% CI 0.11 to 1.53). For knee function, isokinetic training (IKT) had the highest-ranking probability (SUCRA 87.7), but pairwise differences among active modalities were not statistically significant, indicating no clear evidence of clinical superiority. For QoL, eccentric training (ET) ranked first (SUCRA 87.4) and improved QoL versus CON (SMD 1.31; 95% CI 0.01 to 2.63).

**Conclusion:**

Post-ACLR resistance prescription should be outcome-oriented. BFRT may be considered when the primary target is recovery of quadriceps strength and mass, particularly compared with usual standard multimodal rehabilitation. IKT may be considered when aiming to improve knee function, but current evidence does not confirm its superiority over other active modalities. ET may be considered when improving QoL is a major rehabilitation goal, although this finding should be interpreted cautiously given the limited certainty of evidence. These findings should be understood as comparisons between modality-dominant programs and standard multimodal rehabilitation, and may help inform individualized rehabilitation planning following ACLR.

**Systematic review registration:**

https://www.crd.york.ac.uk/prospero/, identifier CRD420251182456.

## 1 Introduction

Anterior cruciate ligament (ACL) injury is common in young, physically active populations and is frequently managed with anterior cruciate ligament reconstruction (ACLR) to restore knee stability and allow return to pivoting sport (Filbay and Grindem, 2019). Recent nationwide data indicate that ACL injury remains a substantial clinical burden. In the United States, the cumulative incidence of ACL tears was 75.19 per 100,000 person-years between 2010 and 2020, and although the annual incidence declined to 53.32 per 100,000 person-years in 2020, the relative utilization of ACL reconstruction increased over the same period (Bergstein et al., 2025). Strength deficits in the quadriceps and hamstrings often persist beyond the early postoperative period and relate to functional performance during the transition to return to sport. Long−term consequences include impaired work participation, health economic costs, and reductions in health−related quality of life (QoL) after traumatic knee injury (Girdwood et al., 2025). Debate continues regarding the timing and criteria for return to sport, reflecting heterogeneity in recovery and the importance of objective benchmarks rather than time alone.

Exercise−based rehabilitation is the cornerstone of postoperative care after ACLR, and contemporary guidelines emphasize progressive load to restore strength, neuromuscular capacity, and limb symmetry in criteria−based pathways (Kotsifaki et al., 2023). Within this paradigm, resistance training is central and can be delivered through distinct modalities. Blood flow restriction training (BFRT) permits low−load exercise under partial vascular occlusion to attenuate atrophy when high loads are not yet tolerated (Gopinatth et al., 2025). Eccentric training (ET) targets high force production during lengthening to stimulate hypertrophy and tendon-muscle adaptation. Isokinetic training (IKT) allows patients to perform maximal voluntary effort at a constant angular velocity, providing accommodating resistance throughout the range of motion (Wang et al., 2023). Plyometric training (PT) applies the stretch-shortening cycle to rebuild explosive capacity and landing control in the late phase of rehabilitation (Buckthorpe and Della Villa, 2021). These approaches differ in biological stimulus, equipment requirements, and typical phase of implementation, which makes comparative evaluation clinically relevant. In practice, these modalities may also be prescribed within open or closed kinetic chain configurations according to symptoms, graft tolerance, neuromuscular control, and rehabilitation stage. Recent evidence suggests that open and closed kinetic chain exercises have comparable effects on knee pain, laxity, quadriceps strength, and function after ACL rehabilitation, indicating that kinetic chain selection and resistance modality are complementary components of exercise prescription rather than competing strategies (Pamboris et al., 2024).

An increasing number of randomized controlled trials (RCTs) have evaluated distinct resistance-training modalities following ACLR. Evidence suggests that perioperative BFRT can mitigate early postoperative atrophy and enhance short-term strength recovery, although these effects may diminish over longer follow-up (Okoroha et al., 2023). IKT programs have shown benefits for strength, proprioception, and neuromuscular control compared with standard rehabilitation, while late-phase PT regimens improve explosive strength and dynamic stability during return-to-sport training (Wang et al., 2023; Li et al., 2025). Conventional meta-analyses support these findings but remain limited by small sample sizes, methodological heterogeneity, and inconsistent reporting of intervention parameters (Gopinatth et al., 2025). Several quantitative syntheses have demonstrated that BFR yields modest advantages in quadriceps hypertrophy and strength relative to non-BFR training, yet the overall certainty of evidence is moderate to low due to variability in cuff pressure, training load, and duration. Beyond BFR-focused reviews, most systematic analyses pool heterogeneous exercise programs, obscuring differences between specific resistance-training approaches. Although previous reviews have addressed broader rehabilitation strategies, including kinetic chain configuration, no prior network meta-analysis has comprehensively contrasted individual resistance modalities after ACLR (Zhong et al., 2025). This evidence gap highlights the need for a unified comparative framework to guide precision exercise prescription in postoperative rehabilitation.

Network meta−analysis (NMA) permits simultaneous comparison of multiple interventions by combining direct and indirect evidence within a single coherent model, and provides probabilistic rankings through the surface under the cumulative ranking curve (SUCRA) (Leucht et al., 2016). The present study conducts an NMA of RCTs to compare BFRT, ET, IKT, and PT against standard multimodal rehabilitation programs without a dominant experimental modality and against one another, focusing on quadriceps strength, knee function, quadriceps mass, and QoL to inform exercise prescription and clinical decision making in postoperative care after ACLR.

## 2 Methods

This systematic review and network meta-analysis followed the Preferred Reporting Items for Systematic Reviews and Meta-Analyses (PRISMA) 2020 statement and its extension for network meta-analyses (Hutton et al., 2015; Page et al., 2021). The methodological framework adhered to the Cochrane Handbook for Systematic Reviews of Interventions. The review was registered with PROSPERO (CRD420251182456).

### 2.1 Literature search and information sources

A systematic search was performed in PubMed, Embase, the Cochrane Central Register of Controlled Trials (CENTRAL), Web of Science, and Google Scholar, covering publications from inception to 30 September 2025. The search strategy incorporated both Medical Subject Headings (MeSH) and free-text terms, combining relevant concepts such as “Anterior Cruciate Ligament Reconstruction,” “Exercise Therapy,” “Resistance Training,” “Isokinetic Training,” “Eccentric Exercise,” “Blood Flow Restriction,” and “Plyometric Training.” Boolean operators “AND” and “OR” were used to combine search terms appropriately.

**Figure 1:**
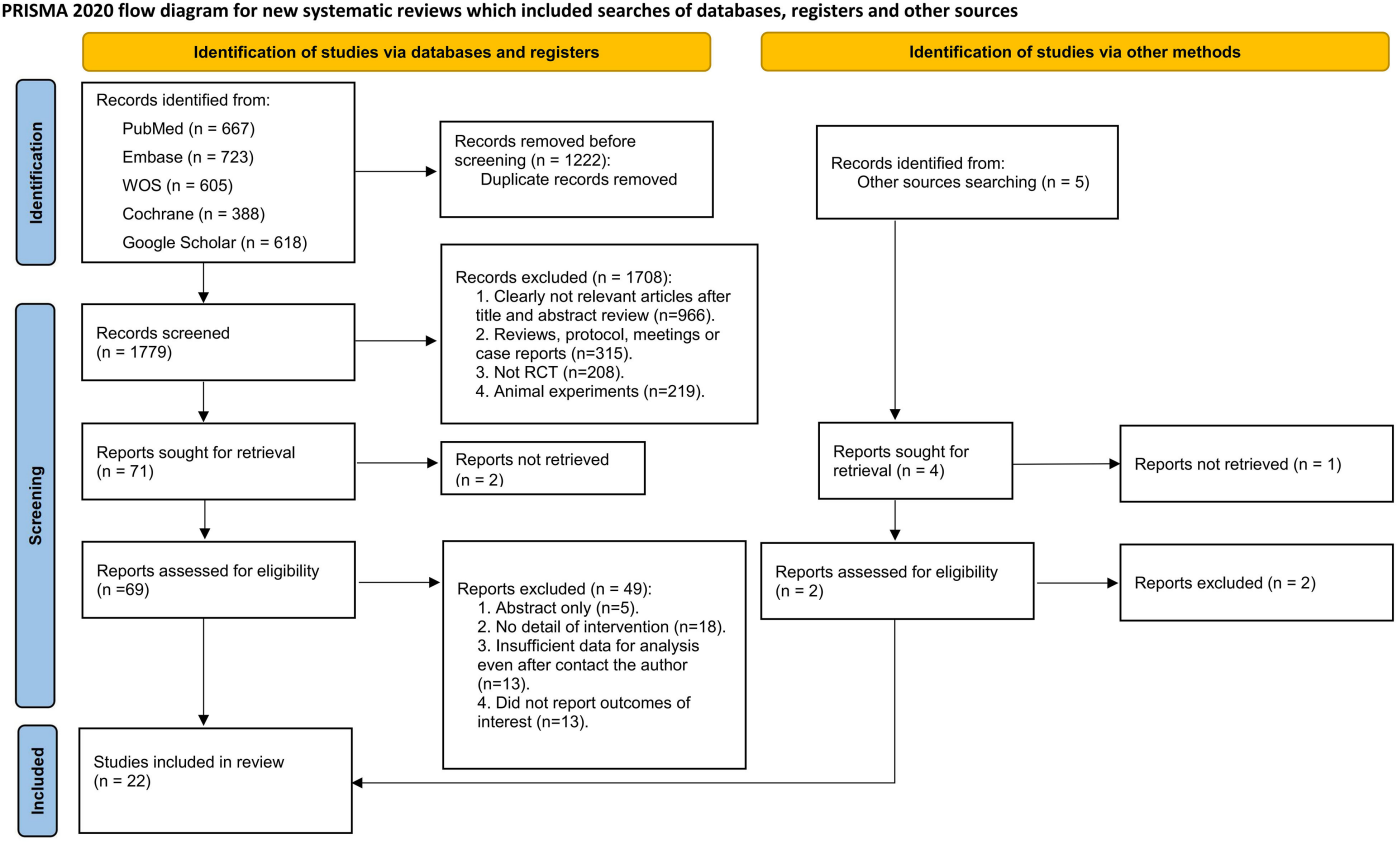
PRISMA flow diagram of study identification, screening, eligibility assessment, and inclusion.

The complete search syntax for each database is provided in **Supplementary Material 1**. Reference lists of included trials and related systematic reviews were manually examined to capture any additional eligible studies. Records identified through these supplementary methods were classified in **Figure 1** as records identified by manual searching of reference lists and related reviews. Two reviewers independently conducted the search and screening; any disagreement was settled by consensus or third-party adjudication.

### 2.2 Eligibility criteria

Studies were included if they satisfied the following criteria: (a) Participants: adults or adolescents who underwent primary ACLR, regardless of sex, age, graft type, or baseline activity level; (b) Interventions: structured resistance-based training programs, including eccentric training, isokinetic training, blood-flow restriction training, or plyometric training; (c) Comparators: standard multimodal post-operative rehabilitation programs without a dominant experimental resistance modality, or other exercise-based interventions; (d) Outcomes: at least one of the following—quadriceps strength, quadriceps cross-sectional area or volume, knee function scores, or health-related quality of life; and (e) Study design: RCTs published in English and subjected to peer review.

Studies were excluded if they met any of the following conditions: (a) participants had revision or multi-ligament reconstruction, severe cartilage injury, or additional lower limb pathology; (b) the intervention did not primarily involve a clearly classifiable resistance modality of interest, or combined non-exercise elements that could confound outcomes; multimodal neuromuscular rehabilitation programs were not excluded *a priori*, but were classified as comparator rehabilitation when no single resistance modality predominated; (c) insufficient details were provided regarding intervention frequency, intensity, or duration; (d) data for primary outcomes were unavailable or could not be obtained after contacting authors four times over six weeks; (e) the study design was non-randomized, quasi-experimental, or only presented as an abstract; and (f) the study involved animal or cadaveric models.

Two reviewers independently applied these criteria to all retrieved records. Any inconsistencies were resolved by discussion or, when necessary, through a senior reviewer’s decision.

### 2.3 Data extraction and management

Eligible trials were organized and de-duplicated using EndNote X9. Data extraction was carried out independently by two investigators using a standardized form. Extracted information included: (a) general study details (authors, year, and study design); (b) participant characteristics (sample size, age, sex distribution, and BMI); (c) intervention features (training modality, intensity, frequency, session duration, total intervention period, timing relative to surgery, BFRT cuff pressure or occlusion level, and IKT angular velocity when available); (d) comparator information; and (e) outcome variables, including the timing of outcome assessment relative to the intervention and any follow-up assessment beyond the intervention period, when reported.

If studies reported non-standard statistics, means and standard deviations were estimated from medians, interquartile ranges, or confidence intervals following Cochrane conversion methods (Higgins and Green, 2008). When multiple arms used the same intervention, results were combined to prevent double-counting. Missing data were requested from corresponding authors up to four times within six weeks. All extracted data were cross-checked for accuracy and recorded in **Supplementary Material 2**.

### 2.4 Risk of bias assessment

Two reviewers independently evaluated the methodological quality of each RCT using the Cochrane Risk of Bias 2 (RoB 2) tool, assessing five domains: randomization process, deviations from intended interventions, missing outcome data, measurement of outcomes, and selection of reported results (Sterne et al., 2019). Each domain was judged as “low risk,” “some concerns,” or “high risk.” Disagreements were resolved by discussion or adjudication by a third author. The overall bias level was summarized for each study.

### 2.5 Classification of interventions and network construction

To ensure conceptual clarity and transitivity within the network, interventions were categorized according to their dominant resistance characteristics: (a) eccentric training (ET), emphasizing controlled muscle lengthening under mechanical load to enhance tensile strength and hypertrophy; (b) isokinetic training (IKT), allowing maximal effort through constant angular velocity to facilitate uniform load across the range of motion; (c) blood flow restriction training (BFRT), applying controlled external compression during low-load resistance exercise to partially restrict blood flow and stimulate muscle growth; (d) plyometric training (PT), promoting explosive strength through rapid stretch-shortening cycles; and (e) standard multimodal rehabilitation (CON), consisting of routine post-surgical programs for range-of-motion recovery, progressive strengthening, and, where applicable, neuromuscular or functional exercise, but without a single dominant experimental modality such as BFRT, IKT, ET, or PT.

Each intervention and comparator was consistently coded across studies. A network diagram was generated to visualize connections among interventions. The assumptions of transitivity and similarity were verified through assessment of participant characteristics, intervention delivery, and outcome definitions before performing the quantitative synthesis. Accordingly, CON was interpreted as a reference category of usual multimodal rehabilitation rather than a no-exercise or no-resistance control. Because many CON protocols included progressive strengthening, neuromuscular exercise, or functional training, comparisons with CON should be understood as comparisons against standard mixed rehabilitation practice rather than against the absence of resistance-based exercise.

### 2.6 Outcome measures

Primary outcomes included quadriceps muscle strength and knee function. Strength was measured using isokinetic or isometric dynamometry, reported as peak torque (Nm/kg). Secondary outcomes included quadriceps muscle size measured by magnetic resonance imaging or ultrasonography, as well as functional scores obtained from validated instruments such as the Lysholm Knee Score, International Knee Documentation Committee (IKDC) form, Western Ontario and McMaster Universities Osteoarthritis Index (WOMAC), and Cincinnati Knee Rating Scale. Health-related quality of life, when reported, was extracted from tools such as the SF-36.

### 2.7 Statistical analysis

All statistical analyses were conducted using Stata version 17.0 (StataCorp LLC, College Station, TX, USA). For continuous outcomes, standardized mean differences (SMDs) and 95% confidence intervals (CIs) were computed. Between-study heterogeneity was evaluated using the I^2^ statistic, with values of 25%, 50%, and 75% representing low, moderate, and high heterogeneity, respectively.

A frequentist random-effects network meta-analysis framework was applied using the network and mvmeta packages. Between-study variance was estimated through the restricted maximum likelihood (REML) method. Network consistency was assessed globally through the design-by-treatment interaction model and locally through node-splitting analysis. A P-value below 0.05 indicated statistically significant inconsistency.

Relative treatment ranking was estimated using surface under the cumulative ranking curve (SUCRA) values, with higher values indicating superior relative performance. Because SUCRA is a ranking summary rather than a direct effect estimate, these values were interpreted descriptively and in conjunction with pairwise SMDs and their 95% CIs. Pairwise comparisons and ranking probabilities were summarized in league tables. In each lower-triangle cell, the reported value represents the SMD with 95% CI and p value for the column-defining intervention relative to the row-defining intervention. Positive values favor the column intervention, whereas negative values favor the row intervention. Potential publication bias and small-study effects were explored through comparison-adjusted funnel plots and Egger’s test, with supplementary trim-and-fill analysis performed where feasible. Given the limited number of studies for some outcomes, these results were interpreted cautiously. Exploratory univariable meta-regression analyses were additionally performed where sufficient direct comparisons were available to examine whether selected intervention parameters modified treatment effects. Prespecified moderators included BFRT timing and BFRT cuff-pressure category. Because only three IKT studies were available and angular-velocity protocols were heterogeneous, IKT angular velocity was summarized descriptively rather than modeled inferentially. Predictive interval plots were generated to evaluate the dispersion and robustness of pooled estimates (Chaimani et al., 2013). All analyses were two-sided, with P < 0.05 considered statistically significant. In addition, for the QoL outcome, a leave-one-out sensitivity analysis was performed by sequentially excluding one study at a time from the QoL dataset. Between-study heterogeneity for this supplementary analysis was quantified using the I^2^ statistic. The certainty of evidence for each network comparison was assessed using the GRADE framework adapted for network meta-analysis. The assessment considered within-study bias, reporting bias, indirectness, imprecision, heterogeneity, and incoherence. Evidence certainty was rated as high, moderate, low, or very low. Comparisons relying mainly on indirect evidence, sparse direct evidence, wide confidence intervals, or unstable estimates were downgraded accordingly. The comparison-level GRADE assessment is provided in **Supplementary File 9**.

## 3 Results

### 3.1 Characteristics of included studies

The comprehensive literature search initially identified 3001 records across all databases. After removing 1222 duplicates, 1779 unique articles remained for title and abstract screening. A total of 1708 studies were excluded at this stage for failing to meet inclusion criteria, leaving 71 full-texts for detailed assessment. Following eligibility evaluation, 22 RCTs involving 797 participants were included in the final NMA (**Figure 1**) (Ohta et al., 2003; Tagesson et al., 2008; Kınıklı et al., 2014; Grapar Zargi et al., 2016; Iversen et al., 2016; Friedmann-Bette et al., 2018; Hughes et al., 2019; Curran et al., 2020; Nambi et al., 2020; Vidmar et al., 2020; Kacin et al., 2021; Milandri and Sivarasu, 2021; Oliveira et al., 2022; Vieira de Melo et al., 2022; Kasmi et al., 2023a; Khalil et al., 2023; Okoroha et al., 2023; Tepa et al., 2024; Erickson et al., 2025; Ohlsen et al., 2025; Patra et al., 2025; Sevinc et al., 2025).

The included trials were published between 2003 and 2025, with a median publication year of 2021, reflecting a notable increase in research interest during the past decade. The studies were conducted in 14 countries, most frequently in the United States (n = 5), Brazil (n = 3), Slovenia (n = 2), and Turkey (n = 2). The total sample size per study ranged from 10 to 96 participants, with a median of 32 participants per trial. The mean age of participants ranged from 15.7 to 41.7 years (median 26.2 years). Baseline BMI was reported in 14 studies, varying between 21.6 and 26.2 kg/m² (median 24.2 kg/m²). Among all included studies, one was a three-arm trial, while the remaining 21 adopted a two-arm parallel design.

In terms of intervention characteristics, 12 studies implemented BFRT, 8 applied ET, 3 used IKT, and 2 incorporated PT. Most control groups (n = 20) employed standard multimodal rehabilitation (CON), rather than a no-exercise or no-resistance control, as the comparator. These CON programs commonly included elements such as range-of-motion exercise, progressive strengthening, neuromuscular training, or functional exercise, but did not contain a single dominant experimental modality such as BFRT, IKT, ET, or PT. Across studies, training frequency ranged from 2 to 6 sessions per week, with intervention durations varying from 4 to 24 weeks. Timing relative to surgery was heterogeneous, spanning different postoperative phases. For the purpose of network classification, each trial was coded according to the dominant experimental modality delivered during the reported intervention period, although background rehabilitation commonly continued across groups. Outcome assessment was performed predominantly at the end of the intervention period, whereas longer-term retention follow-up was available in only a limited number of studies and was not sufficiently consistent for formal synthesis. The detailed study characteristics, including intervention protocols, adherence rates, and outcome measures, are summarized in **Supplementary File 2**.

### 3.2 Results of the network meta-analysis

#### 3.2.1 Quadriceps strength

**Figure 2:**
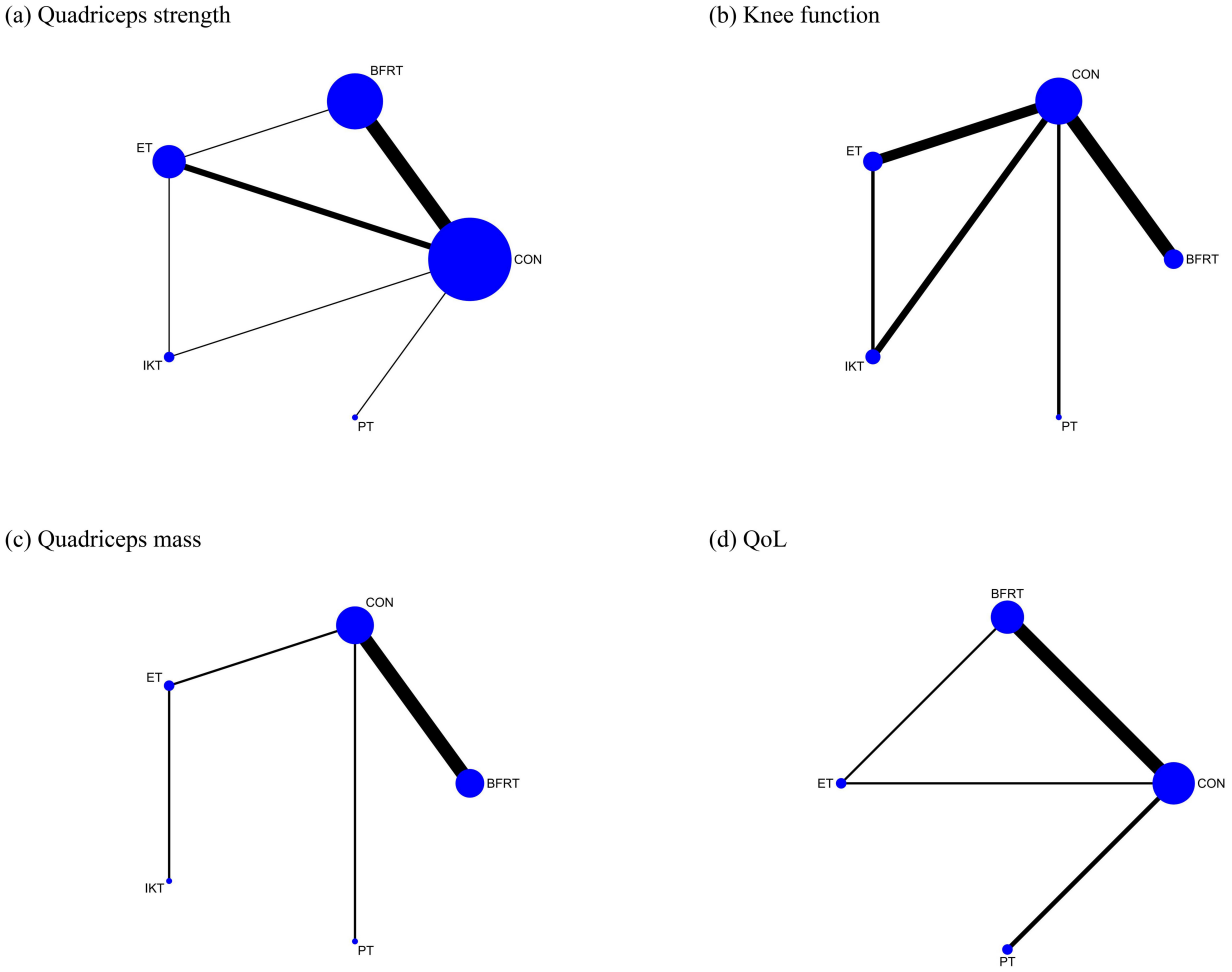
Network plots of resistance training modalities after ACL reconstruction for: **(a)** quadriceps strength, 18 studies; **(b)** knee function, 10 studies; **(c)** quadriceps mass, 10 studies; and **(d)** quality of life, 7 studies. Each node represents an intervention category, including BFRT, ET, IKT, PT, and CON. Node size reflects the amount of evidence contributing to each intervention, and line thickness reflects the number of direct comparisons between interventions.

18 RCTs involving 693 participants evaluated the comparative effects of resistance-based interventions on quadriceps strength after ACLR. The treatment network included direct and indirect comparisons across BFRT, ET, IKT, PT, and CON, as illustrated in **Figure 2a**. The network geometry demonstrated adequate interconnectivity among the interventions, supporting transitivity assumptions.

**Figure 3:**
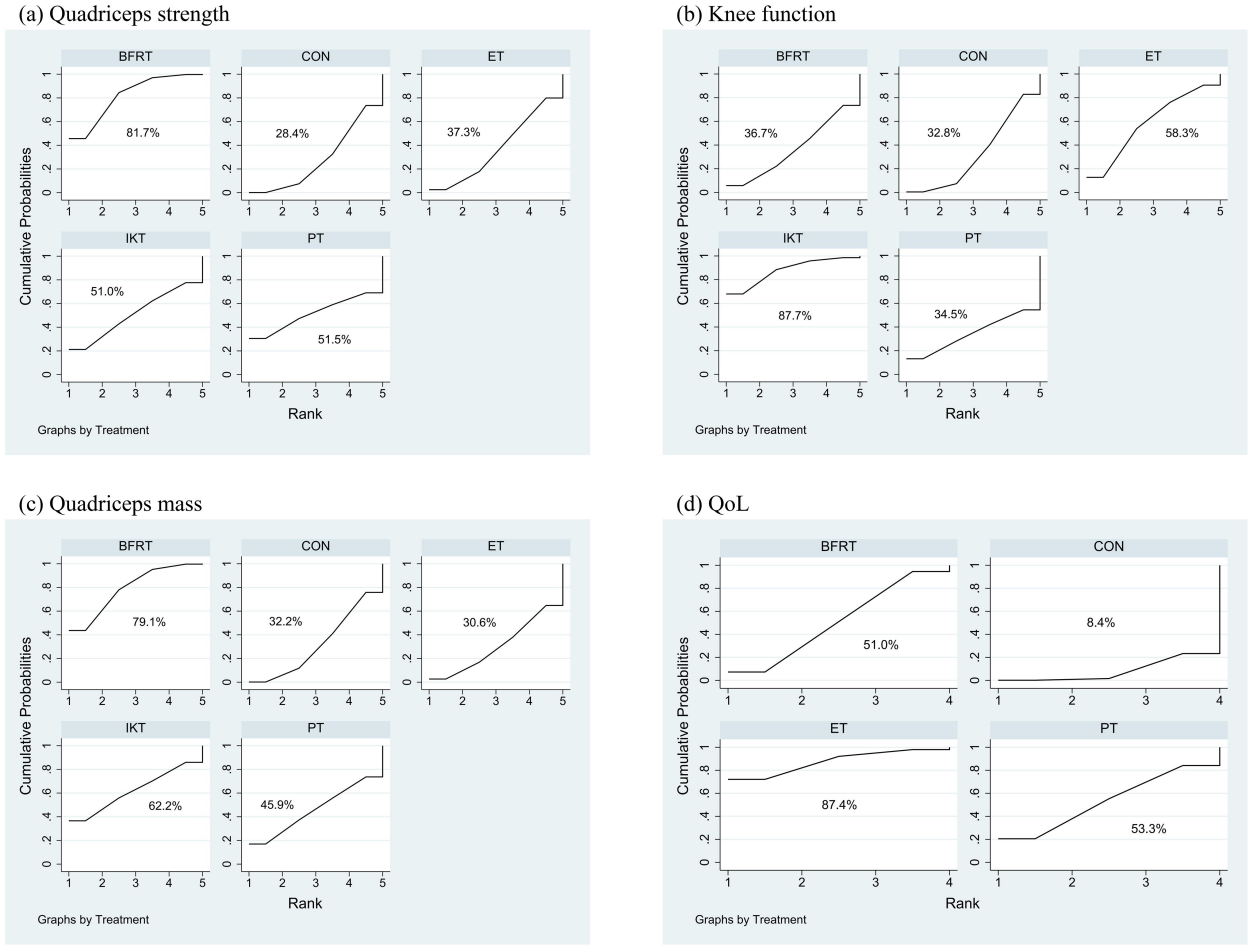
SUCRA probability ranking plots of resistance training modalities after ACL reconstruction for: **(a)** quadriceps strength; **(b)** knee function; **(c)** quadriceps mass; and **(d)** quality of life. Higher SUCRA values indicate a greater probability of ranking among the more effective interventions for a given outcome. These rankings are probabilistic summaries and should be interpreted together with pairwise effect estimates, confidence intervals, and GRADE certainty, rather than as definitive evidence of superiority.

According to the SUCRA ranking (**Figure 3a**), based on the 18 studies included for this outcome, the three most effective interventions for improving quadriceps strength were BFRT (81.7%), PT (51.5%), and IKT (51.0%), whereas CON (28.4%) ranked lowest. Quantitatively, as shown in **Table 1**, BFRT produced a statistically significant improvement compared with CON (SMD = 0.40, 95% CI: 0.07-0.73). No significant differences were detected among other resistance training modalities.

**Table 1:** League tables of quadriceps strength of resistance training modalities after ACL reconstruction.

Intervention	BFRT	PT	IKT	ET	CON
BFRT	BFRT				
PT	0.24 (-0.92, 1.39), p = 0.684	PT			
IKT	0.25 (-0.57, 1.07), p = 0.550	0.01 (-1.33, 1.35), p = 0.988	IKT		
ET	0.35 (-0.14, 0.84), p = 0.162	0.11 (-1.06, 1.29), p = 0.854	0.10 (-0.67, 0.87), p = 0.799	ET	
CON	0.40 (0.07, 0.73), p = 0.018	0.17 (-0.94, 1.27), p = 0.763	0.15 (-0.61, 0.92), p = 0.701	0.05 (-0.35, 0.45), p = 0.806	CON

Each lower-triangle cell shows the standardized mean difference (SMD), 95% confidence interval (CI), and p value for the column-defining intervention relative to the row-defining intervention. Positive values favor the column intervention, whereas negative values favor the row intervention.

The predictive interval plots showed the direction of effect consistently favored BFRT, and no evidence of inconsistency between direct and indirect estimates was observed in node-splitting analyses (p > 0.05).

#### 3.2.2 Knee function

10 studies including 339 participants assessed knee function outcomes. The evidence network for this endpoint is presented in **Figure 2b**, showing multiple closed loops among BFRT, ET, IKT, PT, and CON.

The SUCRA ranking (**Figure 3b**) suggested that IKT had the highest-ranking probability for knee function (87.7%), followed by ET (58.3%) and BFRT (36.7%), whereas CON (32.8%) ranked lowest. However, as shown in **Table 2**, pairwise comparisons among active interventions were not statistically significant (all p > 0.05). Therefore, the ranking results should be interpreted as relative probability rather than evidence of clinical superiority.

**Table 2:** League tables of knee function of resistance training modalities after ACL reconstruction.

Intervention	IKT	ET	BFRT	PT	CON
IKT	IKT				
ET	1.45 (-1.60, 4.50), p = 0.351	ET			
BFRT	2.32 (-1.25, 5.88), p = 0.202	0.87 (-2.38, 4.11), p = 0.599	BFRT		
PT	2.65 (-2.63, 7.94), p = 0.326	1.20 (-3.87, 6.28), p = 0.643	0.34 (-4.70, 5.37), p = 0.895	PT	
CON	2.35 (-0.41, 5.12), p = 0.096	0.90 (-1.44, 3.24), p = 0.451	0.04 (-2.22, 2.30), p = 0.972	-0.30 (-4.80, 4.20), p = 0.896	CON

Each lower-triangle cell shows the standardized mean difference (SMD), 95% confidence interval (CI), and p value for the column-defining intervention relative to the row-defining intervention. Positive values favor the column intervention, whereas negative values favor the row intervention.

Consistency testing through the design-by-treatment interaction model showed no evidence of global inconsistency (p = 0.64), and local inconsistency was minimal.

#### 3.2.3 Quadriceps mass

10 trials with a total of 293 participants evaluated quadriceps mass. The network diagram depicting direct and indirect evidence is shown in **Figure 2c**.

SUCRA results (**Figure 3c**) indicated that BFRT (79.1%), IKT (62.2%), and PT (45.9%) ranked as the most effective modalities for increasing quadriceps muscle mass, whereas CON (32.2%) and ET (30.6%) ranked lowest. As summarized in **Table 3**, BFRT significantly increased quadriceps mass compared with CON (SMD = 0.82, 95% CI: 0.11-1.53).

**Table 3:** League tables of quadriceps mass of resistance training modalities after ACL reconstruction.

Intervention	BFRT	IKT	PT	CON	ET
BFRT	BFRT				
IKT	0.24 (-2.21, 2.69), p = 0.848	IKT			
PT	0.62 (-1.17, 2.41), p = 0.497	0.38 (-2.49, 3.25), p = 0.795	PT		
CON	0.82 (0.11, 1.53), p = 0.024	0.58 (-1.77, 2.93), p = 0.629	0.20 (-1.44, 1.85), p = 0.812	CON	
ET	0.93 (-0.90, 2.76), p = 0.319	0.69 (-0.95, 2.32), p = 0.408	0.31 (-2.04, 2.66), p = 0.796	0.11 (-1.58, 1.79), p = 0.898	ET

Each lower-triangle cell shows the standardized mean difference (SMD), 95% confidence interval (CI), and p value for the column-defining intervention relative to the row-defining intervention. Positive values favor the column intervention, whereas negative values favor the row intervention.

Predictive interval plots confirmed the robustness of this finding, with effect estimates consistently favoring BFRT across study subgroups. No evidence of local or global inconsistency was detected (all p > 0.05).

#### 3.2.4 QoL

7 studies comprising 173 participants assessed QoL. The treatment network and data contribution across interventions are displayed in **Figure 2d**.

Based on SUCRA probabilities (**Figure 3d**), ET (87.4%), PT (53.3%), and BFRT (51.0%) demonstrated the greatest likelihood of improving QoL, while CON (8.4%) showed the lowest ranking. As shown in **Table 4**, ET significantly improved QoL compared with CON (SMD = 1.31, 95% CI: 0.01-2.63). Because the QoL analysis included only a small number of studies, an additional sensitivity analysis was performed. In the supplementary direct-comparison analysis, between-study heterogeneity was substantial (I^2^ = 73.5%). In leave-one-out analyses, the pooled SMD ranged from 0.29 to 0.69, and heterogeneity remained moderate to high (I^2^ = 68.5% to 77.2%) (**Supplementary File 8**). These findings indicate that the direction of effect was generally stable, but the precision of the estimate was sensitive to omission of individual small studies. Therefore, the QoL findings should be interpreted cautiously.

**Table 4:** League tables of quality of life (QoL) of resistance training modalities after ACL reconstruction.

Intervention	ET	PT	BFRT	CON
ET	ET			
PT	0.68 (-1.14, 2.51), p = 0.465	PT		
BFRT	0.75 (-0.55, 2.06), p = 0.260	0.07 (-1.39, 1.53), p = 0.925	BFRT	
CON	1.31 (0.01, 2.63), p = 0.050	0.63 (-0.63, 1.88), p = 0.325	0.56 (-0.18, 1.29), p = 0.135	CON

Each lower-triangle cell shows the standardized mean difference (SMD), 95% confidence interval (CI), and p value for the column-defining intervention relative to the row-defining intervention. Positive values favor the column intervention, whereas negative values favor the row intervention.

Visual inspection of comparison-adjusted funnel plots revealed slight asymmetry. No significant inconsistency was detected in node-splitting analysis (p = 0.57).

#### 3.2.5 Exploratory analysis of intervention parameters

Exploratory study-level meta-regression was performed for BFRT comparisons using timing and cuff-pressure category as moderators. For quadriceps strength, neither BFRT timing nor cuff-pressure category significantly modified the pooled effect (timing: β = -0.14, 95% CI -1.12 to 0.84, p = 0.751; cuff-pressure category: β = 0.20, 95% CI -0.58 to 0.97, p = 0.579). For quadriceps mass, larger effects were observed numerically in studies using later-phase or higher-pressure BFRT, but these associations were not statistically significant (timing: β = 1.66, 95% CI -0.59 to 3.91, p = 0.110; cuff-pressure category: β = 1.18, 95% CI -0.63 to 3.00, p = 0.144). Because only three IKT studies were available and their angular-velocity protocols were heterogeneous, formal meta-regression for IKT was not considered reliable and was therefore summarized descriptively in the **Supplementary File 7**.

### 3.3 Risk of bias and publication bias

Across the 22 included RCTs, overall methodological quality was rated as high to moderate. Specifically, 14 studies were judged as low risk of bias, and 8 as having some concerns. For the randomization process, 17 trials were low risk and 5 presented some concerns. Regarding deviations from intended interventions, 19 were low risk and 3 were some concerns. For missing outcome data, 21 were low risk and 1 was some concerns. In the measurement of outcomes, 20 were low risk and 2 had some concerns. For selective reporting, all 22 were rated low risk. A summary of bias assessments for each domain is provided in **Supplementary File 3**.

Potential publication bias was assessed using comparison-adjusted funnel plots, Egger’s test, and supplementary trim-and-fill analysis (**Supplementary Files 4**, **6**). The funnel plots showed mild asymmetry for some outcomes, suggesting possible small-study effects. Egger’s tests were not statistically significant across outcomes; however, these findings were interpreted cautiously because several analyses were based on a limited number of studies. The supplementary trim-and-fill analysis did not materially alter the overall direction of the pooled effects, suggesting that potential publication bias was unlikely to substantially change the main conclusions.

### 3.4 Certainty of evidence

The comparison-level GRADE assessment is summarized in **Supplementary File 9**. For quadriceps strength, BFRT versus CON was rated as moderate certainty, whereas BFRT versus ET and ET versus CON were rated as low certainty, and the remaining comparisons were rated as very low certainty because of indirectness, imprecision, and sparse direct evidence. For quadriceps mass, BFRT versus CON was also rated as moderate certainty, while all other comparisons were rated as very low certainty. For knee function, all comparisons were rated as very low certainty, mainly due to imprecision, sparse networks, and limited direct evidence between active modalities. For QoL, all comparisons were rated as very low certainty because of the small number of studies, substantial heterogeneity, and imprecision. These findings suggest that the most reliable evidence supports BFRT compared with conventional rehabilitation for quadriceps recovery, whereas ranking-based findings for knee function and QoL should be interpreted cautiously.

## 4 Discussion

This network meta-analysis synthesized evidence from 22 randomized controlled trials including 797 individuals after ACL reconstruction to compare commonly used resistance training modalities. Overall, the findings suggest an outcome-specific pattern of benefit rather than a single universally optimal approach. BFRT showed the most consistent evidence for quadriceps strength and muscle mass recovery. IKT had the highest-ranking probability for knee function, but pairwise contrasts among active modalities were not statistically significant, indicating that this ranking should be interpreted as a relative probability signal rather than evidence of clinical superiority. ET ranked highest for QoL, with PT and BFRT also showing probabilities of benefit. Compared with previous BFRT-focused reviews and broader exercise therapy reviews, the present NMA extends the literature by comparing modality-dominant resistance programs within a single network framework, while also distinguishing resistance modality from kinetic chain configuration (Pamboris et al., 2024; Gopinatth et al., 2025; Zhong et al., 2025). However, GRADE certainty differed substantially across outcomes. Moderate certainty evidence was observed only for BFRT versus CON in quadriceps strength and quadriceps mass, whereas most comparisons involving knee function, QoL, IKT, and PT were rated as low or very low certainty. Therefore, these findings should be interpreted as conditional, outcome-oriented guidance rather than definitive evidence of superiority among active modalities.

Quadriceps restoration is a primary rehabilitation objective after ACLR because extensor torque supports shock absorption, deceleration, landing control, and symmetrical limb function. The present findings regarding BFRT are broadly consistent with previous randomized evidence and pairwise meta-analyses showing early to mid-term gains in quadriceps strength and hypertrophy after ACL reconstruction (Colapietro et al., 2023; Li et al., 2025). While previous BFRT-focused reviews mainly compared BFRT with non-BFRT or standard rehabilitation, our network approach suggests that BFRT remains the most consistently supported modality for quadriceps recovery when evaluated alongside ET, IKT, and PT. BFRT permits low-load resistance exercise under individualized external cuff pressure, which may be useful when higher joint loads are not yet tolerated (Cerqueira et al., 2021). Its effects are biologically plausible, as partial occlusion increases metabolic stress, promotes fast-twitch motor unit recruitment at low loads, and supports anabolic signaling related to hypertrophy and force development (Geng et al., 2024). This may be particularly relevant after ACLR, when pain, effusion, and arthrogenic quadriceps inhibition limit voluntary activation and tolerance to heavy loading. The low external load may also reduce joint shear and discomfort, supporting early adherence (Patterson et al., 2019). Accordingly, BFRT may be considered a targeted option for early quadriceps recovery, with progression to higher-load training as tissue tolerance improves. Protocol heterogeneity in occlusion pressure, cuff width, set structure, and timing still underscores the need for standardized dosing and careful screening for contraindications (de Queiros et al., 2024).

Knee function is another critical rehabilitation outcome after ACLR because it integrates strength, proprioception, neuromuscular control, and dynamic stability. IKT showed the highest-ranking probability for knee function, but the absence of significant pairwise differences indicates that current evidence does not support clear superiority over other active modalities. This is consistent with previous literature reporting mixed findings regarding resistance modalities for functional recovery (Kasmi et al., 2023a). Compared with broader ACL rehabilitation reviews that often treat exercise therapy as a combined category, the present NMA suggests that functional recovery cannot be attributed to resistance modality alone. IKT may support controlled torque production at a constant angular velocity, ET may improve deceleration and landing control through loaded muscle lengthening, and BFRT may facilitate early muscle recovery when high-load training is not tolerated (Kasmi et al., 2021; Wang et al., 2023; Li et al., 2025). These modalities may therefore address different components of knee function rather than form a definitive hierarchy. Exercise specificity, movement velocity, kinetic chain configuration, neuromuscular control demands, rehabilitation stage, and progression strategy may also influence functional outcomes. Accordingly, the high-ranking probability for IKT should be interpreted as hypothesis-generating, particularly because all knee function comparisons were rated as very low certainty by GRADE.

Health-related QoL remains an important patient-centered outcome after ACLR because it reflects physical recovery, confidence, emotional well-being, and return to meaningful activities. The finding that ET may be favorable for QoL is consistent with previous literature suggesting that eccentric-focused rehabilitation may improve perceived recovery, movement confidence, and functional performance (Kasmi et al., 2021; Kasmi et al., 2023b). Because ET emphasizes controlled muscle lengthening under load, it may support braking, landing, and directional control, which are clinically relevant to confidence in the reconstructed knee. PT and BFRT may also contribute to QoL through different pathways, with PT supporting explosive neuromuscular control in later rehabilitation and BFRT allowing low-load strengthening when symptoms or loading tolerance limit conventional exercise. However, the QoL finding should be interpreted cautiously because the evidence base was small and heterogeneous, leave-one-out sensitivity analyses suggested limited robustness, and GRADE rated QoL comparisons as very low certainty. Therefore, ET may be considered when patient-reported recovery is a major rehabilitation goal, but this finding remains provisional.

From a clinical perspective, these findings support a phase-oriented rather than uniform application of resistance modalities after ACLR. BFRT may be considered in the early postoperative phase when pain, effusion, and limited load tolerance restrict conventional strengthening. IKT may be introduced in the mid-rehabilitation phase when symptoms and range of motion permit structured torque training. ET or PT may be more relevant later, when rehabilitation shifts toward deceleration, landing control, and return-to-sport performance. These modalities should not be interpreted as mutually exclusive, because ACLR rehabilitation commonly progresses across phases and may include different modalities over time. The present NMA classified studies according to the dominant modality during a defined intervention window rather than a complete rehabilitation pathway. Thus, these suggestions should be viewed as practical considerations rather than fixed prescriptions. BFRT should also be preceded by appropriate clinical screening and used cautiously, or avoided when indicated, in patients with peripheral vascular disease, increased thrombotic risk, or other relevant vascular conditions. Because joint symptoms, loading tolerance, and anterior knee laxity are important in early rehabilitation, modality selection should also consider graft tolerance, movement quality, and kinetic chain configuration. BFRT, ET, IKT, and PT can be adapted within open or closed kinetic chain exercises according to rehabilitation goals. Recent evidence suggests that appropriately prescribed open and closed kinetic chain exercises have comparable effects on knee pain, anterior knee laxity, quadriceps strength, and function after ACL rehabilitation (Pamboris et al., 2024). Therefore, the present findings should inform resistance stimulus selection within a broader criteria-based rehabilitation pathway rather than replace clinical decisions regarding kinetic chain configuration or joint loading.

This study has two notable strengths. First, it isolates and compares discrete resistance modalities after ACL reconstruction using a network meta-analytic framework restricted to randomized controlled trials. This design allows coherent integration of direct and indirect evidence across BFRT, ET, IKT, PT, and CON, with outcome-specific rankings for quadriceps strength, quadriceps mass, knee function, and QoL. Second, methodological rigor was high, including PRISMA-NMA reporting, RoB 2 assessment, transitivity and consistency evaluation, REML random-effects modeling, predictive intervals, and assessment of small-study effects. Several limitations should be considered. First, the network was uneven for some contrasts and outcomes. PT and IKT were represented by few trials, limiting precision and reducing power for head-to-head comparisons. Second, intervention and measurement heterogeneity may have introduced residual confounding that could not be modeled with study-level data. Protocols varied in BFRT cuff pressures, cuff widths, loading schemes, set structures, functional outcome instruments, and follow-up windows. Time since surgery, graft characteristics, and kinetic chain configuration were also inconsistently reported, precluding more detailed subgroup or meta-regression analyses. Third, CON represented usual multimodal rehabilitation rather than a true no-resistance control. Therefore, the findings should be understood as comparisons between modality-dominant programs and standard multimodal rehabilitation practice, not as comparisons between resistance training and no training. Fourth, the synthesis relied on study-level summary statistics and SMDs across heterogeneous scales, which may complicate clinical interpretation. Pain, joint loading tolerance, and anterior knee laxity were not included as network outcomes, limiting direct comparison of safety and symptom response across modalities. Language restriction to English and the possibility of selective reporting remain additional sources of uncertainty, although Egger’s tests were not significant and funnel asymmetry was slight. Most outcomes were assessed immediately after intervention, and evidence on longer-term maintenance was limited. Finally, most treatment rankings were supported by low or very low certainty evidence and should not be interpreted as definitive hierarchies. Clinical recommendations should therefore remain conditional until larger head-to-head trials become available.

## 5 Conclusion

This network meta-analysis suggests outcome-specific differences among resistance modalities after ACL reconstruction. BFRT may be considered for improving quadriceps strength and muscle mass compared with standard multimodal rehabilitation, with moderate certainty evidence for these comparisons. IKT showed the highest-ranking probability for knee function, but no significant pairwise differences were observed among active modalities and the certainty of evidence was very low. ET may be considered for health-related QoL, although this finding remains provisional. Overall, resistance prescription after ACLR should be individualized and outcome-oriented, and treatment rankings should not be interpreted as definitive hierarchies of effectiveness, particularly for sparsely studied modalities such as IKT and PT, where rankings were informed by few trials and indirect comparisons. Modality selection should be integrated into criteria-based rehabilitation, patient tolerance, kinetic chain configuration, and clinical safety considerations.

## Data Availability

The original contributions presented in the study are included in the article/**Supplementary Material**. Further inquiries can be directed to the corresponding author.
