# Supplementary material for: Comparative effectiveness of resistance training modalities after anterior cruciate ligament reconstruction: a network meta-analysis of randomized controlled trials
Source: Front Physiol. 2026 Aug 3;17:1792091. doi: 10.3389/fphys.2026.1792091 (PMC13477874; doi:10.3389/fphys.2026.1792091)
Supplement: Supplementary file 1 [file DataSheet1.docx]

**Supplementary**

Table of Contents:

**[Supplementary 1: Search Strategy	2](#_Toc159076731)**

[Supplementary 2: Characteristics of studies and subjects included in the review	5](#_Toc159076743)

[Supplementary 3: Risk of Bias](#_Toc159076747)	18

[Supplementary 4: Funnel Plots of Publication Bias](#_Toc159076747)	20

[Supplementary 5: Forest Plots](#_Toc159076747)	22

[Supplementary 6: Trim-and-fill analysis](#_Toc159076747)	24

[Supplementary 7: Meta-regression of selected intervention parameters](#_Toc159076747)	25

[Supplementary 8: Leave-one-out sensitivity analysis for QoL](#_Toc159076747)	26

[Supplementary 9: Grade of outcomes](#_Toc159076747)	27

# Supplementary 1: Search Strategy

***Search Strategy:***

| #7 | Search: (((((((anterior cruciate ligament[MeSH Terms]) OR (Anterior Cruciate Ligament Reconstruction[MeSH Terms])) OR ("Anterior Cruciate Ligament Reconstruction"[Title/Abstract])) OR ("ACL reconstruction"[Title/Abstract])) OR ("Anterior Cruciate Ligament"[Title/Abstract])) AND (( "Exercise"[Mesh] OR "Exercise Therapy"[Mesh] OR "Resistance Training"[Mesh] OR "Yoga"[Mesh] OR "Tai Ji"[Mesh] OR "Dance Therapy"[Mesh] OR "Muscle Stretching Exercises"[Mesh] OR "Weight-Bearing"[Mesh] OR "Physical Therapy Modalities"[Mesh] OR rehabilitation[tiab] OR "physical therap*"[tiab] OR physiotherap*[tiab] OR exercise*[tiab] OR train*[tiab] OR "physical activit*"[tiab] OR "strength training"[tiab] OR resist*[tiab] OR "muscle strengthening"[tiab] OR isokinetic*[tiab] OR isometric*[tiab] OR eccentric*[tiab] OR concentric*[tiab] OR ("open kinetic chain"[tiab] OR "closed kinetic chain"[tiab] OR OKC[tiab] OR CKC[tiab]) OR "electrical stimulation"[tiab] OR NMES[tiab] OR ("blood flow restriction"[tiab] OR BFR[tiab] OR KAATSU[tiab] OR "occlusion training"[tiab]) OR ("balance training"[tiab] OR "proprioceptive training"[tiab] OR "neuromuscular training"[tiab] OR "postural training"[tiab] OR "dual-task training"[tiab] OR perturbation[tiab] OR plyometric*[tiab] OR "sensorimotor training"[tiab]) OR ("aquatic therapy"[tiab] OR hydrother*[tiab] OR "water exercise"[tiab] OR "water-based exercise"[tiab] OR "aquatic exercis*"[tiab]) OR yoga[tiab] OR tai chi[tiab] OR taiji[tiab] OR qigong[tiab] OR pilates[tiab] OR dance[tiab] OR "dance training"[tiab] OR "dance therapy"[tiab] OR "vibration training"[tiab] OR ("mind-body exercise"[tiab] OR "mind body exercise"[tiab]) OR ("early rehabilitation"[tiab] OR "accelerated rehabilitation"[tiab]) OR "aerobic exercise*"[tiab] OR "endurance training"[tiab] ))) AND ((( "Quadriceps Muscle"[Mesh] OR quadriceps[tiab] OR "knee extensor*"[tiab] OR "thigh muscle*"[tiab] ) AND ( "Muscle Strength"[Mesh] OR "Muscle Contraction"[Mesh] OR "Torque"[Mesh] OR strength[tiab] OR "muscle strength"[tiab] OR torque[tiab] OR "peak torque"[tiab] OR MVC[tiab] OR "maximal voluntary contraction"[tiab] OR "maximal voluntary"[tiab] OR isokinetic[tiab] OR isometric[tiab] OR concentric[tiab] OR eccentric[tiab] OR dynamometry[tiab] OR dynamometer[tiab] OR "rate of force development"[tiab] OR RFD[tiab] OR "limb symmetry index"[tiab] OR LSI[tiab] OR "functional performance"[tiab] OR "neuromuscular function"[tiab] OR "knee extensor strength"[tiab] )) OR (( "Quadriceps Muscle"[Mesh] OR quadriceps[tiab] OR "knee extensor*"[tiab] OR "thigh muscle*"[tiab] ) AND ( "Muscle, Skeletal"[Mesh] OR "Muscle Strength"[Mesh] OR "Muscle, Skeletal/anatomy and histology"[Mesh] OR "Muscle, Skeletal/diagnostic imaging"[Mesh] OR "muscle mass"[tiab] OR "lean mass"[tiab] OR "muscle size"[tiab] OR "muscle volume"[tiab] OR "muscle cross-sectional area"[tiab] OR "cross-sectional area"[tiab] OR CSA[tiab] OR "muscle thickness"[tiab] OR thickness[tiab] OR "muscle hypertrophy"[tiab] OR hypertrophy[tiab] OR "muscle atrophy"[tiab] OR atrophy[tiab] OR morphology[tiab] OR architecture[tiab] OR "muscle composition"[tiab] )))) AND ((randomized controlled trial[pt] OR controlled clinical trial[pt] OR randomized[tiab] OR placebo[tiab] OR drug therapy[sh] OR randomly[tiab] OR trial[tiab] OR groups[tiab]) NOT (animals[mh] NOT humans[mh])) |
| --- | --- |
| #6 | Search: (randomized controlled trial[pt] OR controlled clinical trial[pt] OR randomized[tiab] OR placebo[tiab] OR drug therapy[sh] OR randomly[tiab] OR trial[tiab] OR groups[tiab]) NOT (animals[mh] NOT humans[mh]) |
| #5 | Search: (( "Quadriceps Muscle"[Mesh] OR quadriceps[tiab] OR "knee extensor*"[tiab] OR "thigh muscle*"[tiab] ) AND ( "Muscle Strength"[Mesh] OR "Muscle Contraction"[Mesh] OR "Torque"[Mesh] OR strength[tiab] OR "muscle strength"[tiab] OR torque[tiab] OR "peak torque"[tiab] OR MVC[tiab] OR "maximal voluntary contraction"[tiab] OR "maximal voluntary"[tiab] OR isokinetic[tiab] OR isometric[tiab] OR concentric[tiab] OR eccentric[tiab] OR dynamometry[tiab] OR dynamometer[tiab] OR "rate of force development"[tiab] OR RFD[tiab] OR "limb symmetry index"[tiab] OR LSI[tiab] OR "functional performance"[tiab] OR "neuromuscular function"[tiab] OR "knee extensor strength"[tiab] )) OR (( "Quadriceps Muscle"[Mesh] OR quadriceps[tiab] OR "knee extensor*"[tiab] OR "thigh muscle*"[tiab] ) AND ( "Muscle, Skeletal"[Mesh] OR "Muscle Strength"[Mesh] OR "Muscle, Skeletal/anatomy and histology"[Mesh] OR "Muscle, Skeletal/diagnostic imaging"[Mesh] OR "muscle mass"[tiab] OR "lean mass"[tiab] OR "muscle size"[tiab] OR "muscle volume"[tiab] OR "muscle cross-sectional area"[tiab] OR "cross-sectional area"[tiab] OR CSA[tiab] OR "muscle thickness"[tiab] OR thickness[tiab] OR "muscle hypertrophy"[tiab] OR hypertrophy[tiab] OR "muscle atrophy"[tiab] OR atrophy[tiab] OR morphology[tiab] OR architecture[tiab] OR "muscle composition"[tiab] )) |
| #4 | Search: ( "Quadriceps Muscle"[Mesh] OR quadriceps[tiab] OR "knee extensor*"[tiab] OR "thigh muscle*"[tiab] ) AND ( "Muscle Strength"[Mesh] OR "Muscle Contraction"[Mesh] OR "Torque"[Mesh] OR strength[tiab] OR "muscle strength"[tiab] OR torque[tiab] OR "peak torque"[tiab] OR MVC[tiab] OR "maximal voluntary contraction"[tiab] OR "maximal voluntary"[tiab] OR isokinetic[tiab] OR isometric[tiab] OR concentric[tiab] OR eccentric[tiab] OR dynamometry[tiab] OR dynamometer[tiab] OR "rate of force development"[tiab] OR RFD[tiab] OR "limb symmetry index"[tiab] OR LSI[tiab] OR "functional performance"[tiab] OR "neuromuscular function"[tiab] OR "knee extensor strength"[tiab] ) |
| #3 | Search: ( "Quadriceps Muscle"[Mesh] OR quadriceps[tiab] OR "knee extensor*"[tiab] OR "thigh muscle*"[tiab] ) AND ( "Muscle, Skeletal"[Mesh] OR "Muscle Strength"[Mesh] OR "Muscle, Skeletal/anatomy and histology"[Mesh] OR "Muscle, Skeletal/diagnostic imaging"[Mesh] OR "muscle mass"[tiab] OR "lean mass"[tiab] OR "muscle size"[tiab] OR "muscle volume"[tiab] OR "muscle cross-sectional area"[tiab] OR "cross-sectional area"[tiab] OR CSA[tiab] OR "muscle thickness"[tiab] OR thickness[tiab] OR "muscle hypertrophy"[tiab] OR hypertrophy[tiab] OR "muscle atrophy"[tiab] OR atrophy[tiab] OR morphology[tiab] OR architecture[tiab] OR "muscle composition"[tiab] ) |
| #2 | Search: ("Resistance Training"[Mesh] OR "Strength Training"[tiab] OR "Resistance Exercise"[tiab] OR "eccentric training"[tiab] OR "eccentric exercise"[tiab] OR "isokinetic training"[tiab] OR "isokinetic exercise"[tiab] OR "blood flow restriction"[tiab] OR "BFR"[tiab] OR "KAATSU"[tiab] OR "occlusion training"[tiab] OR "concentric training"[tiab] OR "concentric exercise"[tiab] OR "enhanced training"[tiab] OR "muscle strengthening"[tiab] OR "muscle strengthening exercise"[tiab]) |
| #1 | Search: ((((anterior cruciate ligament[MeSH Terms]) OR (Anterior Cruciate Ligament Reconstruction[MeSH Terms])) OR ("Anterior Cruciate Ligament Reconstruction"[Title/Abstract])) OR ("ACL reconstruction"[Title/Abstract])) OR ("Anterior Cruciate Ligament"[Title/Abstract]) |

# Supplementary 2: Characteristics of studies and subjects included in the review

| **Study** | **Country/Region** | **Study design** | **Subjects**  **(intervention/ control)** | **Sex (male/female)**  **(intervention/ control)** | **Mean age**  **(intervention/ control)** | **BMI** | **Disease** | **Intervention detail** | | **Session duration** | **Training frequency** | **Duration** | **Adhenrence** | **Outcomes** |
| --- | --- | --- | --- | --- | --- | --- | --- | --- | --- | --- | --- | --- | --- | --- |
|  |  |  |  |  |  |  |  | **Intervention group** | **Control group** |  |  |  |  |  |
| Curran et al. (2020) | USA | Open-label RCT | 34 (18/16) | 10/8 vs 5/11 | 15.7 ± 1.3 vs 17.4 ± 3.5 | NR | ACL reconstruction (entering the intensive training phase 10 weeks after surgery) | High-intensity leg extension exercises (concentric or eccentric) combined with BFRT | High-intensity leg extension exercises (concentric or eccentric) | NA | 2 times/week | 8 weeks | 100% | Quadriceps strength, QoL |
| Oliveira et al. (2022) | Brazil | Single-blind RCT | 88 (44/44) | 28/16 vs 31/13 | 28.0 ± 9.48 vs 29.0 ± 9.37 | 23.7 ± 9.33 vs 23.5 ± 8.54 | ACL reconstruction (unilateral and bilateral, entering the resistance training phase 4 months after surgery ) | Strength training, unilateral, lunge (with the supporting leg in front); Leg press; Stretch at the end; Leg extension machine training; Straight leg raise | Strength training, bilateral, squat; leg press; end-range stretch (with low pulley assisting knee extension); leg extension machine training; straight leg raise | NA | 2 times/week | 8 weeks | 39/44 vs 40/44 | Quadriceps strength, Knee function |
| Okoroha et al. (2023) | USA | Open-label RCT | 46 (22/24) | 12/10 vs 16/8 | 25.4 ± 10.6 vs 27.5 ± 12.0 | 24.9 ± 3.1 vs 26.9 ± 5.3 | ACL reconstruction (unilateral,2 weeks after the first visit, and starting from 3 days after the surgery, the treatment was carried out for a period of 3 months) | Perioperative blood flow restriction (BFR) training, combining prehabilitation and postoperative rehabilitation with pneumatic cuffs at 80% limb occlusion pressure applied during resistance and functional exercises. | Identical prehabilitation and postoperative rehabilitation program without BFR application. | NA | Before the operation:  5 times/week After the operation:  2-3 times/week | 14 weeks | 16/22 vs 22/24 | Quadriceps strength, Quadriceps mass |
| Sevinc et al. (2024) | Turkey | Single-blind RCT | 24 (13/11) | 13/0 vs 11/0 | 24.69 ± 6.99 vs 25.27 ± 5.74 | 24.10 ± 2.35 vs 23.93 ± 2.46 | ACL reconstruction (unilateral, entering the intensive training phase 4-12 weeks after surgery) | Eccentric cross-education training on the uninjured limb combined with blood flow restriction at 80% limb occlusion pressure. | Eccentric cross-education training on the uninjured limb without blood flow restriction. | NA | 2 times/week | 9 weeks | 100% | Quadriceps strength, Quadriceps mass |
| Friedmann-bette et al. (2018) | Germany | Single-blind RCT | 37 (21/16) | 21/0 vs 16/0 | 24 ± 4 vs 26 ± 5 | NR | ACL reconstruction (unilateral, entering the intensive training phase 12 weeks after surgery) | Progressive one-legged concentric/eccentric strength training with eccentric overload (CON/ECC⁺) on an isokinetic leg-press device. | Conventional concentric/eccentric strength training (CON/ECC) on a standard leg-press without eccentric overload. | NA | 2 times/week | 12 weeks | 100% | Quadriceps strength |
| Hughes et al.（2019） | US | Single-blind RCT | 24 (12/12) | 7/5 vs 10/2 | 29 ± 7 vs 29 ± 7 | 25.40 ± 3.86 vs 26.41 ± 4.35 | ACL reconstruction (unilateral, Exercise intervention began 2 weeks after the surgery.) | Blood flow restriction resistance training (BFR-RT) at 30% 1RM during ACL rehabilitation. | Basic rehabilitation (early post-operation + at home: core balance, hip-knee resistance training, functional training), start unilateral leg lifting training two weeks after surgery | 30 min | Family rehabilitation:  3 times/week Single-leg lifting exercise: 2 times/week | 8 weeks | 100% | Quadriceps strength, Knee function, QoL |
| Iversen et al. (2016) | Norway | Single-blind RCT | 24 (12/12) | 7/5 vs 7/5 | 24.9 ± 7.4 vs 29.8 ± 9.3 | NR | ACL reconstruction (unilateral, Using the hamstring tendon graft, exercise intervention was carried out 2 days after the operation.) | Received intermittent blood flow restriction during low-load quadriceps exercises after ACL reconstruction. | Performed the same exercises without occlusion stimulus. | 40 min | 2 times/day | 14 days | NA | Quadriceps mass, QoL |
| Msc et al. (2022) | Brazil | Open-label RCT | 24 (12/12) | 8/4 vs 9/3 | 41.1 ± 9.8 vs 39.6 ± 10.8 | 24.2 ± 3.0 vs 23.6 ± 2.4 | ACL reconstruction (unilateral, Intervention should be initiated in the early postoperative period) | Performed low-load resistance training (30% 1RM) with blood flow restriction at 80% limb occlusion pressure after ACL reconstruction. | Performed the same resistance exercises without occlusion using traditional high-load training (70% 1RM). | NA | 2 times/week | 12 weeks | NA | Quadriceps strength, Knee function, QoL |
| Kasmi et al. (2023) | Tunisia | Open-label RCT | 30 (10/10/10) | 10/0 vs 10/0 vs 10/0 | 20.30 ± 2.83 vs 20.30 ± 2.54 vs 20.4 ± 3.34 | 21.59 ±1.29 vs 21.68 ± 1.65 vs 20.95 ± 1.09 | ACL reconstruction (unilateral, Exercise intervention began 14 weeks after the surgery.) | (a) Eccentric training – performed eccentric quadriceps and hamstring strengthening exercises after ACL reconstruction. (b) Plyometric training – performed jump-based explosive power training. | Followed a traditional rehabilitation program including flexibility, balance, and low-intensity strengthening exercises. | 60 min | 2 times/week | 6 weeks | 100% | Knee function, QoL |
| Kacin et al. (2021) | Slovenia | Single-blind RCT | 18 (6/6/6) | 3/3 vs 3/3 vs 3/3 | 38 ± 6 vs 38 ± 8 vs 36 ± 9 | 26.0 ± 4.9 vs 25.8 ± 5.4 vs 24.8±3.2 | ACL reconstruction (unilateral, Preoperative 3-week exercise intervention.) | Performed low-load resistance exercises for the injured leg (knee extension and flexion at 40 RM to volitional failure) with blood flow restriction applied via a 13.5-cm pneumatic cuff (150 mmHg asymmetric pressure) during each set. | Performed the same low-load resistance exercises with the cuff inflated only to 20 mmHg, providing no actual occlusion stimulus (work-matched sham condition). | NA | 3 times/week | 3 weeks | 100% | Quadriceps strength, Knee function, Quadriceps mass |
| Kinikli et al. (2014) | Turkey | Single-blind RCT | 33 (16/17) | 15/1 vs 16/1 | 33.87 ± 8.19 vs 32.64 ± 8.21 | NR | ACL reconstruction (unilateral, Exercise intervention began 1 weeks after the surgery.) | Performed progressive eccentric and concentric strength training for 12 weeks on a monitored functional squat system (MFSS) starting 3 weeks post-ACL reconstruction, in addition to standard rehabilitation | Followed the standard ACL rehabilitation program without the additional MFSS eccentric–concentric training | NA | 3 times/week | 12 weeks | NA | Quadriceps strength, Knee function |
| Milandri et al. (2021) | South Africa | Open-label RCT | 22 (12/10) | 12/0 vs 10/0 | 25.8 ± 6.4 vs 25.2 ± 6.0 | NR | ACL reconstruction (unilateral, entering the intensive training phase 10-16 weeks after surgery) | Performed progressive eccentric–concentric resistance training using an isoinertial flywheel device, 3 times per week for 8 weeks after ACL reconstruction . | Followed the standard rehabilitation protocol including conventional strength and mobility exercises without flywheel use . | 26 min | 3 times/week | 8 weeks | NA | Quadriceps strength, Quadriceps mass |
| Ohlsen et al. (2025) | USA | Open-label RCT | 10 (5/5) | 6/4 | 26.6 ± 9.8 vs 30.4 ± 10.1 | 22.7 ± 3.5 vs 28.6 ± 5.3 | ACL reconstruction (unilateral, Start the training within 7 to 21 days after the surgery.) | Received blood flow restriction training during standardized postoperative ACL rehabilitation, using 60–80% arterial occlusion pressure for quadriceps and hamstring exercises | Performed the same rehabilitation protocol with sham BFR, applying only ~20% arterial occlusion pressure (non-therapeutic compression) | A total of 200 minutes | 2 times/week | 6 weeks | 100.00% | Knee function, Quadriceps mass, QoL |
| Ohta et al. (2003) | Japan | Open-label RCT | 44 (22/22) | 25/19 | 30 ± 9.7 vs 28 ± 9.7 | NR | ACL reconstruction (unilateral, Exercise intervention began 2 weeks after the surgery.) | Performed low-load resistance training with moderate blood flow restriction using an air tourniquet (≈180 mm Hg) during postoperative rehabilitation for 16 weeks after ACL reconstruction | Performed the same low-load resistance exercises without blood flow restriction throughout the 16-week postoperative program | NA | 6 times/week | 14 weeks | NA | Quadriceps strength, Quadriceps mass |
| Patra et al. (2025) | India | Single-blind RCT | 96 (47/49) | 36/11 vs 39/10 | 29.94 ± 6.78 vs 30.20 ± 7.45 | NR | ACL reconstruction (Exercise intervention began 3 weeks after the surgery.) | Received isoinertial eccentric overload training combined with conventional rehabilitation following ACL reconstruction. | Underwent standard rehabilitation alone without isoinertial training. | IG: 60 min CG: 30 min | Inertial training:  2 times/ week  regular rehabilitation: 6 times/week | 6 weeks | NA | Quadriceps strength |
| Tagesson et al. (2007) | Sweden | Single-blind RCT | 42 (22/20) | 13/9 vs 11/9 | 25 ± 6.25 vs 27 ± 7.25 | NR | ACL reconstruction (unilateral, Intervention was initiated on average 43 days after injury (ranging from 20 to 96 days), without performing surgery.) | Received a comprehensive rehabilitation program supplemented with open kinetic chain (OKC) quadriceps strengthening—seated knee extensions on one leg as the primary exercise, progressing by load and phase | Received the same comprehensive rehabilitation program but with closed kinetic chain (CKC) quadriceps strengthening, mainly single-leg squats | NA | 3 times/week | 16 weeks | NA | Quadriceps strength, Knee function |
| ZARGI et al. (2016) | Slovenia | Single-blind RCT | 20 (10/10) | 16/4 | 33 ± 7 vs 34 ± 10 | 23.7 ± 3.1 vs 23.5 ± 3.9 | ACL reconstruction (unilateral, Start exercising 10 days before the surgery and complete it within 48 hours before the operation.) | Performed knee-extension exercises with blood flow restriction (150 mmHg) using a pneumatic tourniquet. | Performed the same knee-extension exercises with a low-pressure cuff (20 mmHg). | NA | 3 times/week | A total of 5 times | 100% | Quadriceps strength |
| Erickson et al. (2024) | USA | Double-blind RCT | 48 (23/25) | 13/10 vs 15/10 | 21.1 ± 6.3 vs 21.5 ± 5.3 | 24.9 ± 3.9 vs 25.7 ± 4.5 | ACL reconstruction (unilateral, The first intervention began 10 weeks after the injury and one month before the surgery. Standard rehabilitation started 3 days after the surgery, but specific BFRT/fake BFRT training began 2 weeks after the surgery.) | Performed low-load resistance training with pneumatic cuff inflation (60% arterial occlusion pressure) during quadriceps exercises after ACL reconstruction. | Performed identical exercises with sham pressure (20 mmHg) without actual blood flow restriction. | NA | 3 times/week | 20-24 weeks | 93.5 ± 4.6% vs 93.7 ± 4.9% | Quadriceps strength, Quadriceps mass |
| Vidmar et al. (2020) | Brazil | Single-blind RCT | 30 (15/15) | 15/0 vs 15/0 | 26.9 ± 5.8 vs 24.3 ± 4.6 | 23.4 ± 1.0 vs 23.7 ± 1.2 | ACL reconstruction (unilateral, Exercise began approximately 45 days after the surgery.) | Maximal eccentric contractions of the knee extensors were performed on an isokinetic dynamometer at a constant angular velocity of 60°/s. | A constant-load eccentric exercise was performed on a knee extension chair, with the researcher performing the concentric phase and the participant controlling the eccentric phase. | NA | 2 times/week | 6 weeks | NA | Quadriceps strength, Knee function, Quadriceps mass |
| Khalil et al. (2023) | Egypt | Double-blind RCT | 36 (18/18) | 15/3 vs 16/2 | 23.78 ± 3.934 vs 25.22 ± 4.76 | 26.213 ± 3.9471 vs 25.359 ± 2.1013 | ACL reconstruction (unilateral, intervention began 1 week after the surgery.) | Received blood flow restriction training combined with conventional rehabilitation protocol post-ACLR. | Received only conventional rehabilitation protocol post-ACLR. | NA | 3 times/week | 12 weeks | NA | Quadriceps strength |
| Nambi et al. (2020) | Saudi Arabia | Single-blind RCT | 40 (20/20) | 20/0 vs 20/0 | 22.3 ± 1.2 vs 22.9 ± 1.7 | 22.5 ± 1.5 vs 22.6 ± 1.4 | ACL reconstruction (Start of exercise intervention after at least 3 months following ACL injury) | Received isokinetic training for knee muscles using an isokinetic dynamometer at angular velocities of 60°, 90°, and 120°/s, five days per week for 4 weeks. | Completed home-based stretching and strengthening exercises for quadriceps, hamstrings, gluteal, and calf muscles without specialized equipment or progressive load adjustment. | NA | 5 times/week | 4 weeks | NA | Knee function |
| Tepa et al. (2024) | Thailand | Open-label RCT | 27 (14/13) | 12/2 vs 12/1 | 29.8 ± 7.7 vs 28.2 ± 6.8 | 25.4 ± 2.5 vs 23.8 ± 2.9 | ACL reconstruction (unilateral, intervention began during the 6-8 month follow-up after the surgery.) | Received supervised plyometric training twice weekly for 8 weeks, involving jumping and landing exercises (e.g., squat jumps, ankle hops, step-up jumps, and lateral bound stick landings) performed with bodyweight to enhance stretch–shortening cycle activation and neuromuscular control during the late phase of ACL rehabilitation. | Performed resistance training according to ACSM guidelines (75% 1RM, 3 sets of 10 repetitions) using dumbbells and exercises such as squats, lunges, step-ups, and heel raises to strengthen lower limb muscles. | 40 min | 2 times/week | 8 weeks | 100% | Quadriceps strength, Quadriceps mass, QoL |

Note: RCT, randomized controlled trial; ACL, anterior cruciate ligament; BFRT, blood flow restriction training; 1RM, one-repetition maximum; QoL, quality of life; BMI, body mass index; NR, not reported; NA, not available; IG, intervention group; CG, control group. Sex is reported as male/female for both groups. Age and BMI are presented as mean ± SD. Interventions include resistance-based training (eccentric, isokinetic, blood flow restriction). Training frequency is sessions per week, and duration is in minutes. Adherence is given as percentage. Outcomes typically include quadriceps strength, quadriceps mass, knee function, and QoL.

# Supplementary 3: Risk of Bias

| **Author** | **Bias arising from the randomization process** | **Bias due to deviations from intended intervention** | **Bias due to missing outcome data** | **Bias in measurement of the outcome** | **Bias in selection of the reported result** | **Overall** |
| --- | --- | --- | --- | --- | --- | --- |
| Curran et al. (2020) | Low | Low | Low | Low | Low | Low |
| Oliveira et al. (2022) | Low | Low | Low | Low | Low | Low |
| Okoroha et al. (2023) | Some Concerns | Low | Low | Some Concerns | Low | Some Concerns |
| Sevinc et al. (2024) | Some Concerns | Low | Low | Low | Low | Some Concerns |
| Friedmann-bette et al. (2018) | Low | Low | Low | Low | Low | Low |
| Hughes et al.（2019） | Low | Low | Low | Low | Low | Low |
| Iversen et al. (2016) | Low | Low | Low | Low | Low | Low |
| Msc et al. (2022) | Some Concerns | Some Concerns | Low | Low | Low | Some Concerns |
| Kasmi et al. (2023) | Low | Low | Low | Low | Low | Low |
| Kacin et al. (2021) | Some Concerns | Low | Low | Low | Low | Some Concerns |
| Kinikli et al. (2014) | Low | Low | Low | Low | Low | Low |
| Milandri et al. (2021) | Low | Low | Low | Low | Low | Low |
| Ohlsen et al. (2025) | Low | Some Concerns | Low | Some Concerns | Low | Some Concerns |
| Ohta et al. (2003) | Low | Low | Low | Low | Low | Low |
| Patra et al. (2025) | Low | Low | Low | Low | Low | Low |
| Tagesson et al. (2007) | Low | Some Concerns | Low | Low | Low | Some Concerns |
| ZARGI et al. (2016) | Low | Low | Low | Low | Low | Low |
| Erickson et al. (2024) | Low | Low | Low | Low | Low | Low |
| Vidmar et al. (2020) | Low | Low | Some Concerns | Low | Low | Some Concerns |
| Khalil et al. (2023) | Low | Low | Low | Low | Low | Low |
| Nambi et al. (2020) | Low | Low | Low | Low | Low | Low |
| Tepa et al. (2024) | Some Concerns | Low | Low | Low | Low | Some Concerns |

# Supplementary 4: Funnel Plots of Publication Bias

Figure 4.1 The funnel plot of Quadriceps strength. The result of Egger test showed the p=0.523.

Figure 4.2 The funnel plot of Knee function. The result of Egger test showed the p=0.534.

Figure 4.3 The funnel plot of Quadriceps mass. The result of Egger test showed the p=0.398.

Figure 4.4 The funnel plot of QoL. The result of Egger test showed the p=0.739.

# Supplementary 5: Forest Plots

Figure 5.1 The forest plot of Quadriceps strength. The result of inconsistency test showed the p=0.397.

Figure 5.2 The forest plot of Knee function. The result of inconsistency test showed the p=0.734.

Figure 5.3 The forest plot of Quadriceps mass. The result of inconsistency test showed the p=0.410.

Figure 5.4 The forest plot of QoL. The result of inconsistency test showed the p=0.486.

# Supplementary 6: Trim-and-fill analysis

| **Outcome** | **Estimated number of imputed studies** | **Original pooled SMD** | **Trim-and-fill adjusted SMD** | **Interpretation** |
| --- | --- | --- | --- | --- |
| Quadriceps strength | 1 | 0.275 | 0.23 | Slight attenuation; direction unchanged |
| Knee function | 0 | 0.659 | 0.659 | No imputed study; result unchanged |
| Quadriceps mass | 1 | 0.604 | 0.473 | Mild attenuation; direction unchanged |
| Health-related quality of life (QoL) | 1 | 0.536 | 0.424 | Mild attenuation; direction unchanged |

# Supplementary 7: Meta-regression of selected intervention parameters

| **Outcome** | **Moderator** | **Comparisons, n** | **βcoefficient** | **95% CI** | **P value** | **Interpretation** |
| --- | --- | --- | --- | --- | --- | --- |
| Quadriceps strength | BFRT timing, early/perioperative/preoperative vs mid-postoperative | 10 | -0.14 | -1.12 to 0.84 | 0.751 | No significant effect modification |
| Quadriceps strength | BFRT cuff-pressure category, high vs lower or unclear | 10 | 0.2 | -0.58 to 0.97 | 0.579 | No significant effect modification |
| Quadriceps mass | BFRT timing, early/perioperative/preoperative vs mid-postoperative | 6 | 1.66 | -0.59 to 3.91 | 0.11 | Non-significant trend toward larger effects in later-phase protocols |
| Quadriceps mass | BFRT cuff-pressure category, high vs lower or unclear | 6 | 1.18 | -0.63 to 3.00 | 0.144 | Non-significant trend toward larger effects with higher pressure |
| Knee function | IKT angular velocity | 3 | Not modeled | Not applicable | Not applicable | Descriptive only because of sparse and heterogeneous data |

# Supplementary 8: Leave-one-out sensitivity analysis for QoL

| **Omitted study set** | **Pooled SMD** | **95% CI** | **I^2^ (%)** |
| --- | --- | --- | --- |
| Overall QoL | 0.53 | -0.10 to 1.17 | 73.5 |
| Smallest BFRT vs CON study (n = 10) | 0.69 | 0.17 to 1.22 | 68.5 |
| BFRT vs CON study A (n = 24) | 0.43 | -0.25 to 1.10 | 71.9 |
| BFRT vs CON study B (n = 24) | 0.62 | -0.08 to 1.31 | 74.5 |
| BFRT vs CON study C (n = 24) | 0.52 | -0.20 to 1.24 | 76.2 |
| PT vs CON study (n = 27) | 0.59 | -0.12 to 1.30 | 75.5 |
| BFRT vs CON study set (n = 29) | 0.42 | -0.34 to 1.19 | 77.2 |
| BFRT vs ET study (n = 34) | 0.65 | -0.03 to 1.33 | 70.6 |
| ET/PT/CON multiarm study (n = 40) | 0.29 | -0.39 to 0.97 | 70.1 |

# Supplementary 9: Grade of outcomes

Table 6.1 GRADE for Quadriceps Strength

| **Comparison** | **Number of studies** | **Within study bias** | **Reporting bias** | **Indirectness** | **Imprecision** | **Heterogeneity** | **Incoherence** | **Confidence rating** |
| --- | --- | --- | --- | --- | --- | --- | --- | --- |
| BFRT:PT | 0 | No concerns | No concerns | Major concerns | Major concerns | Some concerns | No concerns | Very low |
| BFRT:IKT | 0 | No concerns | No concerns | Major concerns | Major concerns | Some concerns | No concerns | Very low |
| BFRT:ET | 1 | No concerns | No concerns | No concerns | Some concerns | Some concerns | No concerns | Low |
| BFRT:CON | 10 | No concerns | No concerns | No concerns | No concerns | Some concerns | No concerns | Moderate |
| PT:IKT | 0 | No concerns | No concerns | Major concerns | Major concerns | Some concerns | No concerns | Very low |
| PT:ET | 0 | No concerns | No concerns | Major concerns | Major concerns | Some concerns | No concerns | Very low |
| PT:CON | 1 | No concerns | No concerns | No concerns | Major concerns | Some concerns | No concerns | Very low |
| IKT:ET | 1 | No concerns | No concerns | No concerns | Major concerns | Some concerns | No concerns | Very low |
| IKT:CON | 1 | No concerns | No concerns | No concerns | Major concerns | Some concerns | No concerns | Very low |
| ET:CON | 5 | No concerns | No concerns | No concerns | Some concerns | Some concerns | No concerns | Low |

Table 6.2 GRADE for Knee Function

| **Comparison** | **Number of studies** | **Within study bias** | **Reporting bias** | **Indirectness** | **Imprecision** | **Heterogeneity** | **Incoherence** | **Confidence rating** |
| --- | --- | --- | --- | --- | --- | --- | --- | --- |
| IKT:ET | 1 | No concerns | No concerns | No concerns | Major concerns | Some concerns | No concerns | Very low |
| IKT:BFRT | 0 | No concerns | No concerns | Major concerns | Major concerns | Some concerns | No concerns | Very low |
| IKT:PT | 0 | No concerns | No concerns | Major concerns | Major concerns | Some concerns | No concerns | Very low |
| IKT:CON | 2 | No concerns | No concerns | No concerns | Major concerns | Some concerns | No concerns | Very low |
| ET:BFRT | 0 | No concerns | No concerns | Major concerns | Major concerns | Some concerns | No concerns | Very low |
| ET:PT | 1 | No concerns | No concerns | No concerns | Major concerns | Some concerns | No concerns | Very low |
| ET:CON | 3 | No concerns | No concerns | No concerns | Major concerns | Some concerns | No concerns | Very low |
| BFRT:PT | 0 | No concerns | No concerns | Major concerns | Major concerns | Some concerns | No concerns | Very low |
| BFRT:CON | 4 | No concerns | No concerns | No concerns | Major concerns | Some concerns | No concerns | Very low |
| PT:CON | 1 | No concerns | No concerns | No concerns | Major concerns | Some concerns | No concerns | Very low |

Table 6.3 GRADE for Quadriceps Mass

| **Comparison** | **Number of studies** | **Within study bias** | **Reporting_bias** | **Indirectness** | **Imprecision** | **Heterogeneity** | **Incoherence** | **Confidence rating** |
| --- | --- | --- | --- | --- | --- | --- | --- | --- |
| BFRT:IKT | 0 | No concerns | No concerns | Major concerns | Major concerns | Some concerns | No concerns | Very low |
| BFRT:PT | 0 | No concerns | No concerns | Major concerns | Major concerns | Some concerns | No concerns | Very low |
| BFRT:CON | 5 | No concerns | No concerns | No concerns | No concerns | Some concerns | No concerns | Moderate |
| BFRT:ET | 0 | No concerns | No concerns | Major concerns | Major concerns | Some concerns | No concerns | Very low |
| IKT:PT | 0 | No concerns | No concerns | Major concerns | Major concerns | Some concerns | No concerns | Very low |
| IKT:CON | 0 | No concerns | No concerns | Major concerns | Major concerns | Some concerns | No concerns | Very low |
| IKT:ET | 1 | No concerns | No concerns | No concerns | Major concerns | Some concerns | No concerns | Very low |
| PT:CON | 1 | No concerns | No concerns | No concerns | Major concerns | Some concerns | No concerns | Very low |
| PT:ET | 0 | No concerns | No concerns | Major concerns | Major concerns | Some concerns | No concerns | Very low |
| CON:ET | 1 | No concerns | No concerns | No concerns | Major concerns | Some concerns | No concerns | Very low |

Table 6.4 GRADE for Health-Related Quality of Life

| **Comparison** | **Number of studies** | **Within study bias** | **Reporting bias** | **Indirectness** | **Imprecision** | **Heterogeneity** | **Incoherence** | **Confidence rating** |
| --- | --- | --- | --- | --- | --- | --- | --- | --- |
| ET:PT | 1 | No concerns | No concerns | No concerns | Major concerns | Major concerns | No concerns | Very low |
| ET:BFRT | 1 | No concerns | No concerns | No concerns | Major concerns | Major concerns | No concerns | Very low |
| ET:CON | 1 | No concerns | No concerns | No concerns | Major concerns | Major concerns | No concerns | Very low |
| PT:BFRT | 0 | No concerns | No concerns | Major concerns | Major concerns | Major concerns | No concerns | Very low |
| PT:CON | 2 | No concerns | No concerns | No concerns | Major concerns | Major concerns | No concerns | Very low |
| BFRT:CON | 5 | No concerns | No concerns | No concerns | Some concerns | Major concerns | No concerns | Very low |

BFRT, blood flow restriction training; CON, conventional rehabilitation; ET, eccentric training; IKT, isokinetic training; PT, plyometric training; QoL, quality of life.
